# Supplementary material for: Antibacterial Activity and Protection Efficiency of Polyvinyl Butyral Nanofibrous Membrane Containing Thymol Prepared through Vertical Electrospinning
Source: Polymers (Basel). 2021 Apr 1;13(7):1122. doi: 10.3390/polym13071122 (PMC8036783; doi:10.3390/polym13071122)
Supplement: Supplementary file 1 [file polymers-13-01122-s001.pdf]

Supporting Information

Antibacterial activity and protection efficiency of polyvinyl butyral nanofibrous membrane containing Thymol prepared through vertical electrospinning

Wen-Chi Lu,[a,b,+] Ching-Yi Chen,[a,+] Chia-Jung Cho,[a,+,*] Manikandan Venkatesan,[a,+] Wei-Hung Chiang,[c] Yang-Yen Yu,[d] Chen-Hung Lee,[e,*] Rong-Ho Lee,[f] Syang-Peng Rwei,[a] and Chi-Ching Kuo[a,*]

[a] Institute of Organic and Polymeric Materials, Research and Development Center of Smart Textile Technology, National Taipei University of Technology, Taipei 10608, Taiwan

[b] Department of Applied Cosmetology, Lee-Ming Institute of Technology, New Taipei City 243083, Taiwan

[c] Department of Chemical Engineering, National Taiwan University of Science and Technology, Taipei 10607, Taiwan

[d] Department of Materials Engineering, Ming Chi University of Technology, New Taipei City 24301, Taiwan

[e] Division of Cardiology, Department of Internal Medicine, Chang Gung Memorial Hospital-Linkou, Chang Gung University College of Medicine, Tao-Yuan 333, Taiwan.

[f] Department of Chemical Engineering, National Chung Hsing University, Taichung 402, Taiwan

+ W.-C. Lu, C.-Y. Chen, C.-J. Cho, and M. Venkatesan contributed equally to this work

*Author to whom all correspondence should be addressed

Tel.: 886-2-27712171*2407; Fax: 886-2-27317174

Correspondence to: Prof. C.-C. Kuo (E-mail: kuocc@mail.ntut.edu.tw)

Dr. C.-H. Lee (E-mail: chl5265@gmail.com)

Dr. C.-J. Cho (E-mail: ppaul28865@mail.ntut.edu.tw)

Table S1. Parameters of the vertical electrospinning device

Group	Voltage (kV)	Distance[1] (mm)	Flow rate (mL/h)
C	25	110	5
D	25	160	5
E	15	160	2
F	18	160	2

Distance[1]：tip to collector

Table S2. Sample weight per unit area in various preparation methods

	mL	weight of solution (g)	area (cm^2)	weight of solution per 5x5 cm^2 (g)	dry weight per 5x5 cm^2 (g)	weight ratio
Nanofibrous membranes	10.08	8.064	1200	0.168	0.0084	1
Film (5x5 cm^2)	1	0.8	25	0.8	0.0400	4.76

Table S3. Quantitative antibacterial properties of Thymol/PVB nanofibrous membrane against *S. aureus*

Bacterial Strain	*S. aureus* ATCC 6538		Concentration of inoculum (CFU/mL)		1.2×10^5
Thymol：PVB (w:w)	control/sample number of bacteria 0 hr (CFU)	control/sample number of bacteria 18-24 hr (CFU)	growth value on the control (F)	growth value on the sample (G)	antibacterial activity value (A)
Control group	2.4×10^4	7.2×10^6	2.5	-	-
0：1	2.4×10^4	2.9×10^6	-	2.1	0.4
0.2：1	2.3×10^4	1.1×10^4	-	-0.3	2.8
0.4：1	2.3×10^4	2.6×10^2	-	-2.0	4.4
0.6：1	2.1×10^4	< 20	-	-3.1	5.6
0.8：1	2.1×10^4	< 20	-	-3.1	5.6
1：1	1.9×10^4	< 20	-	-3.1	5.6

Table S4. Quantitative antibacterial activity of Thymol/PVB nanofibrous membrane against *K. pneumoniae*

Bacterial Strain	*K. pneumoniae* ATCC 4352		Concentration of inoculum (CFU/mL)		1.6×10^5
Thymol：PVB (w:w)	control/sample number of bacteria 0 hr (CFU)	control/sample number of bacteria 18-24 hr (CFU)	growth value on the control (F)	growth value on the sample (G)	antibacterial activity value (A)
Control group	3.0×10^4	5.0×10^7	3.2	-	-
0：1	3.0×10^4	2.6×10^7	-	2.9	0.3
0.2：1	3.0×10^4	1.3×10^6	-	1.6	1.6
0.4：1	2.7×10^4	< 20	-	-3.2	6.4
0.6：1	2.8×10^4	< 20	-	-3.2	6.4
0.8：1	2.4×10^4	< 20	-	-3.2	6.4
1：1	2.4×10^4	< 20	-	-3.2	6.4

Table S5. Quantitative antibacterial activity of Thymol/PVB nanofibrous membrane against *E. coli*

Bacterial Strain	*E. coli* ATCC 8739		Concentration of inoculum (CFU/mL)		$1.2×10^5$
Thymol：PVB (w:w)	control/sample number of bacteria 0 hr (CFU)	control/sample number of bacteria 18-24 hr (CFU)	growth value on the control (F)	growth value on the sample (G)	antibacterial activity value (A)
Control group	$2.3 × 10^4$	$5.5 × 10^7$	3.4	-	-
0：1	$2.2 × 10^4$	$1.6 × 10^7$	-	2.8	0.5
0.2：1	$2.2 × 10^4$	$1.2 × 10^7$	-	2.7	0.7
0.4：1	$2.3 × 10^4$	$6.0 × 10^6$	-	2.4	1.0
0.6：1	$2.2 × 10^4$	< 20	-	-3.1	6.4
0.8：1	$2.1 × 10^4$	< 20	-	-3.1	6.4
1：1	$2.1 × 10^4$	< 20	-	-3.1	6.4

Table S6. Comparison of antibacterial activity values of Thymol/PVB blenders against three bacteria strains

JIS L 1902 Absorption method	antibacterial activity value		
Thymol：PVB (w : w)	*S. aureus* ATCC 6538	*K. pneumoniae* ATCC 4352	*E. coli* ATCC 8739
0：1	0.4	0.3	0.5
0.2：1	2.8	1.6	0.7
0.4：1	4.4	6.4	1.0
0.6：1	5.6	6.4	6.4
0.8：1	5.6	6.4	6.4
1：1	5.6	6.4	6.4

Table S7. Comparison of PP Spun-Bond and Melt-blown nonwoven fabrics

Commercial mask sample	PP Spun-Bond	PP Spun-Bond	Melt-blown
Layer	Outer	Inner	Middle
Diameter	μm	μm	μm
1	28.635	22.804	2.530
2	23.324	21.260	9.278
3	19.668	19.698	3.400
4	18.439	27.785	3.688
5	20.881	18.439	8.920
6	22.091	25.456	3.736
7	24.166	22.804	4.205
8	22.804	24.003	4.118
9	25.012	20.881	1.709
10	30.463	20.591	3.606
Average diameter	23.548	22.372	4.519
Standard deviation	3.760	2.811	2.528

Table S8. Testing results of PFE and Pressure difference

Group	Time hr	CNS 14755		CNS 14777
		PFE(%)	Inspiratory impedance (mmH_2O)	Pressure difference (mmH_2O/cm^2)
F	1	38.6±1.0	3.7±0.1	1.3±0.1
F	2	51.6±0.5	4.8±0.1	1.9±0.1
F	3	66.9±0.7	6.6±0.3	2.7±0.1

F	4	72.5±0.8	7.7±0.2	2.9±0.1
F	5	76.1±1.2	9.1±0.4	3.3±0.3
F	6	83.2±1.1	10.8±0.2	4.7±0.2

Table S9. PFE and BFE test results of 30 commercial masks

No.	PFE(%)	BFE(%)	No.	PFE(%)	BFE(%)
1	18.45	86.2	16	73.45	99.8
2	30.29	96.0	17	76.69	98.8
3	34.14	89.6	18	78.23	99.3
4	38.68	99.1	19	79.42	99.3
5	50.11	92.0	20	77.45	99.4
6	56.88	98.1	21	79.25	99.4
7	58.65	97.8	22	79.98	99.4
8	59.21	97.9	23	75.76	99.5
9	60.59	99.1	24	76.74	99.5
10	61.60	98.2	25	77.90	99.7
11	69.90	95.9	26	79.80	99.7
12	71.99	98.2	27	77.85	99.8
13	73.49	98.4	28	78.05	99.9
14	72.57	99.3	29	80.01	99.5
15	74.43	99.7	30	80.51	99.6

Table S10. Protection efficiency of Thymol/ PVB antibacterial nanofibrous masks

Group / time	PFE (%)	BFE (%)	Pressure difference mmH_2O/cm^2
F /1 h	38.6	82.0	1.3
F /2 h	51.6	84.6	1.9
F /3 h	66.9	88.1	2.7
F /4 h	72.5	88.9	2.9
F /5 h	76.1	98.6	3.3
F /6 h	83.2	99.4	4.7

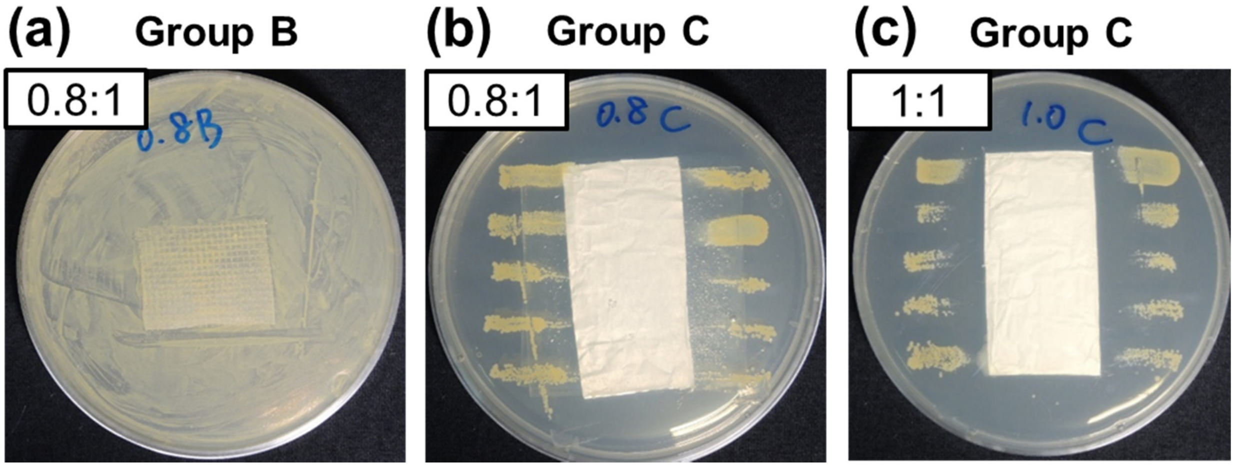

Figure S1. Antibacterial qualitative results of Thymol/PVB nanofibrous membranes on Staphylococcus aureus

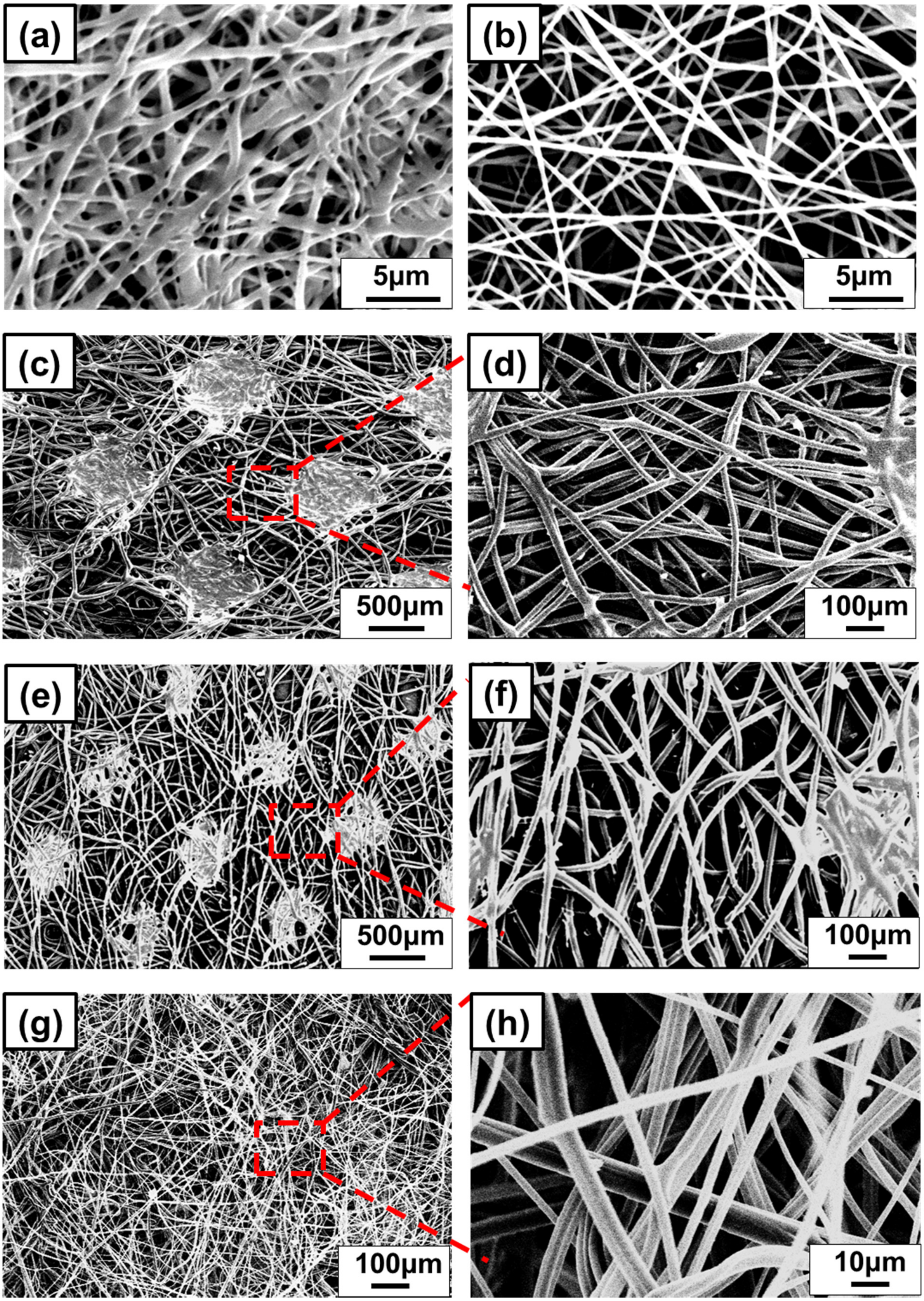

Figure S2. SEM image of the middle layer of the nanofibrous masks (a) D/45 min, (b) E/2 h; SEM image of PP Spun-Bond (c) (d) outer layer, (e) (f) inner layer, (g) (h) Melt-blown nonwoven fabric middle layer.
